# Nanoplastics interfere with plant–mycorrhizal communication and limit plant growth

**DOI:** 10.1093/ismejo/wrag101

**Published:** 2026-04-23

**Authors:** Han Hao Li, Xun Wen Chen, Ming Ge Xing, Yong Xi Zhao, Miao Miao Zhang, Quan Ying Cai, Hui Li

**Affiliations:** Department of Ecology, College of Life Science and Technology, Jinan University, Guangzhou 510632, Guangdong Province, China; MOE Key Laboratory of Tumor Molecular Biology, College of Life Science and Technology, Jinan University, Guangzhou 510632, Guangdong Province, China; Department of Ecology, College of Life Science and Technology, Jinan University, Guangzhou 510632, Guangdong Province, China; MOE Key Laboratory of Tumor Molecular Biology, College of Life Science and Technology, Jinan University, Guangzhou 510632, Guangdong Province, China; Department of Ecology, College of Life Science and Technology, Jinan University, Guangzhou 510632, Guangdong Province, China; MOE Key Laboratory of Tumor Molecular Biology, College of Life Science and Technology, Jinan University, Guangzhou 510632, Guangdong Province, China; Department of Ecology, College of Life Science and Technology, Jinan University, Guangzhou 510632, Guangdong Province, China; MOE Key Laboratory of Tumor Molecular Biology, College of Life Science and Technology, Jinan University, Guangzhou 510632, Guangdong Province, China; Department of Ecology, College of Life Science and Technology, Jinan University, Guangzhou 510632, Guangdong Province, China; MOE Key Laboratory of Tumor Molecular Biology, College of Life Science and Technology, Jinan University, Guangzhou 510632, Guangdong Province, China; Department of Ecology, College of Life Science and Technology, Jinan University, Guangzhou 510632, Guangdong Province, China; MOE Key Laboratory of Tumor Molecular Biology, College of Life Science and Technology, Jinan University, Guangzhou 510632, Guangdong Province, China; Department of Ecology, College of Life Science and Technology, Jinan University, Guangzhou 510632, Guangdong Province, China; MOE Key Laboratory of Tumor Molecular Biology, College of Life Science and Technology, Jinan University, Guangzhou 510632, Guangdong Province, China

**Keywords:** global change factors, ecological risk, toxicity, spore germination, hyphal branching, nutrient cycling

## Abstract

More than 80% of land plants form symbiotic relationships with arbuscular mycorrhizal (AM) fungi for nutrient uptake. As emerging soil pollutants, nanoplastics (NPs) accumulate in both crop and AM fungal tissue, posing non-negligible toxicity and health risks. However, whether and how NPs can impact plant–AM fungal partnership throughout the symbiotic process remains poorly understood. Here, using axenic root-fungal culture, fluorescence NP tracking, and real-time symbiotic signal monitoring, we show that during pre-colonization phase, NPs reduced spore germination rates (−48%) due to the NP accumulation on spore surface, hindering symbiotic signal perception. During the colonization phase, NPs entered fungal cells, disrupted organelles, and accelerated hyphal senescence, consequently reducing hyphal branching length (−22%) and secondary spore production (−32%). In real-world soil, inoculation of secondary spores (reproduced under NPs) formed fewer arbuscule structures (−46%) within maize roots with reduced carbon allocation to AM fungus, leading to a lower hyphal length density (HLD) (−24%). During the post-colonization phase, lower HLD impaired the well-known function of phosphorus (P) mineralization by hyphae-enriched bacteria, reduced soil available P (−5.7%) and maize shoot P (−20%), eventually resulting in compromised plant performance. Our findings reveal an integrated yet largely underexplored mechanism of how NPs hinder plant performance by disrupting the dynamic relationship between plants and their symbiotic partners. In a broader context, understanding the alteration of plant-microbial interaction (rather than separately) under emerging stress can provide ecologically relevant implications for sustaining agricultural and terrestrial ecosystems.

## Graphical Abstract

**Graphical Abstract:**
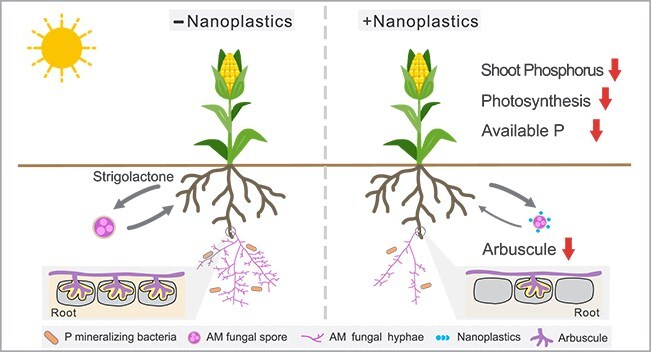

## Introduction

Annual plastic production is projected to reach 1.1 billion tons by 2050 [1]. Plastics are discarded into various ecosystems [2]. In particular, as a major plastic sink, soil receives ∼52% of global plastic pollution [3]. Plastic degradation in soil generates numerous small-sized particles (considered as a factor of global change [4, 5]). Among them, nanoplastics (NPs, <1 μm or 100 nm) have attracted widespread and continuous attention due to their high bioavailability [6]. Previous studies show that small-sized particles (including NPs) can negatively impact individual organisms, such as animals [7, 8], plants [9–11], fungi [12], and bacteria [13, 14]. However, the community ecology of any organisms involves complex pairwise and multipartite interactions, ranging from competition to facilitation (i.e. mutualism) [15, 16]. These biological interactions have profound effects on the sustainability of the ecosystems, such as energy transfer, food webs, and biodiversity regulation [16]. We need to investigate how NPs can affect the interaction between individuals; otherwise, the results would constrain our ecologically relevant understanding. To study the NP effects on biological interactions, the plant–fungal symbiotic system offers a robust avenue. The plant–fungal symbiosis, commonly known as mutualism, is a pillar of biological interactions in terrestrial ecosystems. Particularly, arbuscular mycorrhizal (AM) fungi form symbiotic relationships with >80% of terrestrial plants. They are vital ecosystem regulators linking soil and plant roots, helping host plants acquire essential minerals, particularly phosphorus (P), and resist environmental stresses [17, 18].

The survival of AM fungi is strictly dependent on stable symbiotic establishment with host plants. AM fungi must obtain carbon sources from their plant partners to complete their lifecycle due to the lack of a cytoplasmic fatty acid synthase [19, 20]. This partnership contains multiple phases. During the precolonization phase (including signal perception and spore germination), plant root exudates (particularly strigolactones, SLs) induce calcium (Ca) spiking in fungal spores to promote germination [21]. It has been shown that NPs (100 nm) could adsorb on the surface of fern spores (*Ceratopteris pteridoides*), reducing spore size and spore germination [22]. However, the effects of NPs on the early development and germination of AM fungal spores remain largely unexplored.

After spore germination, fungal hyphae need to enter root cells and form arbuscules (the definitive structures for resource exchange) to establish a functional symbiosis. At the root cell–arbuscule interface, the host plant provides carbon to the fungus and fuels its mitochondrial adenosine triphosphate (ATP) synthesis. This energy drives hyphal elongation, formation of branched absorbing structures (BASs) for P acquisition, and production of secondary spores (reproduction). During such a colonization phase, nanoparticles (~30 and ~200 nm) can enter existing AM fungal hyphae and be transported throughout the entire hyphal network via cytoplasmic streaming, as evidenced by previous studies [23, 24]. NPs within fungal cells can disrupt the integrity of organelles, thereby impairing the development and function of the entire hyphal network. This process raises a key question: how do NPs disrupt the integrity of key organelles, thereby impairing the development and function of the entire hyphal network?

Following the hyphal development, the extraradical hyphal network initiates a subsequent key phase: enabling P mineralization, defined as the postcolonization phase here. Lacking phosphatases and P-solubilizing enzymes to mineralize organic P, AM fungi depend on phosphate-solubilizing bacteria (PSB) to mineralize organic P for uptake and transfer to plants [25]. The extraradical hyphae serve as a transport network for PSB dispersal and a surface for bacterial colonization, thereby enabling the mineralization of organic P for plant uptake. However, unhealthy hyphal development under NP stress may impair the production of metabolites necessary to enrich PSB [26]. Consequently, the capacity of the compromised hyphal networks to mobilize and deliver P to hosts remains unconfirmed.

Corresponding to the above three colonization phases, we hypothesize that (i) NPs can suppress the AM fungal spore germination by interfering with the reception of the germination signals, particularly SLs. (ii) NPs can disrupt the integrity of AM fungal organelles, thereby impairing the extension of the hyphal network and the subsequent secondary spore reproduction. (iii) The compromised hyphal networks take up less P and enrich fewer PSB, so that the P delivered to host plants will be significantly reduced. In this study, to test the first two hypotheses, we cocultured root organs and AM fungus under axenic conditions to investigate the NP effects and corresponding mechanisms on spore germination, hyphal development and activities, and quality of secondary spores. To test the third hypothesis, we examined how NPs can alter the various colonization structures of AM fungi within maize roots and hyphal length in real-world soil. Furthermore, the influence of NPs on soil organic P mineralization, plant P uptake, and plant performance was further analyzed. Collectively, the impacts and mechanisms of NPs on affecting the plant–AM fungal partnership across multiple symbiotic phases have been investigated.

## Materials and methods

### Characterization of nanoplastics

The NPs used were positively charged amine-functionalized polystyrene (PS-NH_2_) because they are more toxic to AM fungi than uncharged and negatively charged NPs [23]. The NPs were purchased from Huge Biotechnology Co., Ltd (Shanghai, China; CAS number 9003-53-6). The NPs were fluorescence-labeled using the nontoxic fluorescein isothiocyanate, which can be visualized under excitation wavelengths of 535/610 nm. Such fluorescence-labeled PS-NH_2_ particles were commonly used as the model plastic particles representing positively charged NPs [23, 27]. The original product suspension contained <0.5% proprietary dispersant/surfactant and <0.1% proprietary preservative (manufacturer’s Material Safety Data Sheet).

The morphology of NPs was characterized using transmission electron microscopy (FEI-Tecnai G2 Spirit TWIN, Thermo) and scanning electron microscopy (SEM, SU8100, Hitachi). The size and Zeta potential (ζ) of NPs were determined with dynamic light scattering (DLS) using a Zetasizer Nano ZS90 instrument (Malvern Instruments Ltd). The PS-NH_2_ NPs had an average hydrodynamic diameter of 25 ± 2.5 nm and a Zeta potential of +45.2 mV. Before the experiment started, all NPs were transferred to a dialysis bag (1 kDa) containing deionized water for 7 d to remove any impurities, with the deionized water being replaced every 12 h. Detailed characterization of the NPs is shown in Fig. S1. We also confirmed that fluorescent dye leakage and quenching of the NPs were negligible during the experimental period (Fig. S2).

### Root organ–arbuscular mycorrhizal fungus culture and nanoplastic treatment

**Figure 1:**
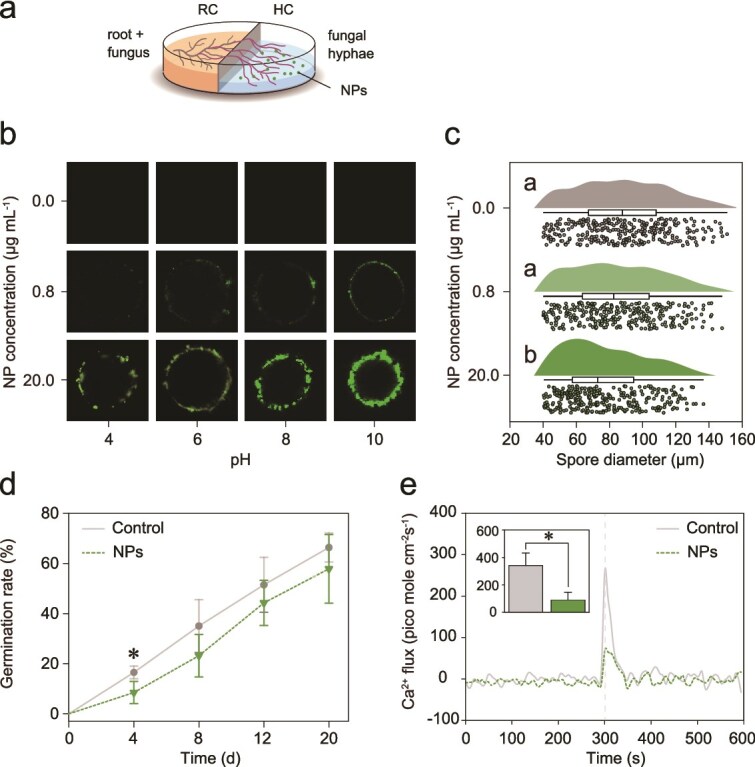
NP distribution on spore surface and NP-treated spore development in root–organ culture. (a) Root organ and AM fungus growth system constructed in a Petri dish. RC, root compartment; HC, hyphal compartment, *n* = 3 culture plates, with 15 spores measured per treatment. (b) NP distribution on the spore surface with various NP concentrations and pH levels. (c) Size distribution of secondary spores in the HC with 0, 0.8, and 20 μg ml^−1^ NPs. *n* = 3 culture plates, with 300 spores measured per treatment. (d) Germination rates of AM fungal spores in culture media. Control, non-NPs; NPs, NPs-treated, *n* = 4 culture plates, with 200 spores measured per treatment. Whiskers are standard deviations. (e) Spore Ca^2+^ spiking in response to the synthetic strigolactone GR24 after exposure to 20 μg ml^−1^ NPs, *n* = 5 culture plates, with five spores measured per treatment. Whiskers in the subplot are standard deviations. Different lowercase letters and asterisks indicate significant differences among/between treatments (ANOVA followed by Tukey’s HSD test or *t-*test, *P* < .05).

To establish root–AM fungal symbiosis, excluding the influence of soil and other microorganisms, we grew hairy roots with AM fungal spores in Petri dishes under axenic conditions (Fig. 1a), as previously described [28]. Detailed procedures are provided in the Supplementary Information (SI).

Fifteen milliliters of modified MSR medium (Table S1, without sucrose and phytogel but containing 0, 0.8, or 20 μg ml^−1^ fluorescently labeled NPs) were added to the HC. Although NP levels in soil are anticipated to increase over time due to continuous degradation of pre-existing plastics [9, 29, 30], rigorous NP quantification in soil pore water remains a major challenge [9]. So, the NP levels selected were based on previous studies testing the effects of NPs on the plant *Arabidopsis* (10–100 μg ml^−1^) [31] and other fungi *Aspergillus* (40 μg ml^−1^) [32]. Also, because the pH of the solution can affect NP attachment/aggregation, we adjusted the pH of the medium with HCl and NaOH (both 0.01 mM) to values as low as 4 and as high as 10. To minimize NP aggregation due to fungal exudates [23], the liquid culture medium containing NPs was renewed every 5 d. All chemicals were purchased from Aladdin Company (China) unless stated otherwise.

### Sample collection and fluorescence detection

After NP treatment for 30 d, hyphae and the secondary spores (after reproduction) were collected using tweezers under a microscope and placed in a 5 ml centrifuge tube containing ultrapure water. These samples were then immediately processed for further analysis. A confocal laser scanning microscope (CLSM, LSM880, Carl Zeiss, Germany) was used to examine the attachment of NPs on the spore surface. The fluorescence intensities were obtained using the Zen 3.0 software (blue edition; Carl Zeiss).

### Secondary spore diameter

The spores collected from the treatment with or without 20 μg ml^−1^ NPs (pH = 5.5) were used to observe the spore diameter. Approximately 300 spores were randomly selected from the HC, centrifuged at 3000 × g for 15 min, and the supernatant was removed. The remaining was ultrasonicated for 2 min to remove NPs that may be attached to the surface of the spores. Afterward, the spores were evenly dispersed in the Petri dish, observed, and photographed using a stereoscope (DFC450, Leica) equipped with a digital camera system. The ImageJ software was used to binarize images and count spores. The highest Feret diameter, as determined by ImageJ software, was used to estimate the spore diameter. Spores smaller than 40 μm in diameter were excluded from the analysis because the ImageJ software readily identifies small-sized air bubbles or contaminants (e.g. hyphal fragments) as spores [33].

### Spore germination rate

Fifty spores were randomly selected to determine the germination rate. The spores were collected in the treatment with or without 20 μg ml^−1^ NPs (medium pH = 5.5). To trigger spore germination without roots, 135 μl (0.1 mM) of the synthetic germination signal (GR24, mimicking SLs) was added to 1 l of autoclaved modified MSR medium [34]. The GR24 was also filtered through a 0.22 μm filter to eliminate bacteria. Fifty spores were transferred to the medium containing GR24 in a sterile environment and solidified by cooling. The Petri dishes were sealed with Parafilm and cultured in a 27°C incubator in the dark. The germination rate of the spores was counted on Days 4, 8, 12, and 20, respectively.

### Analysis of GR24-induced Ca^2+^ signals

The spores collected in the treatment with or without 20 μg ml^−1^ NPs (pH = 5.5) were used. The noninvasive microtest technique (NMT) (BIO-001A, Younger, Barnstable, USA) was used to detect the transient Ca^2+^ signals at the inner spore cell (5 μm from the surface). The spores were immobilized at the bottom of a Petri dish containing 0.1 mM CaCl_2_ and 0.2 mM MES (pH = 5.5), and the determination was started after standing for 30 min. The measurement lasted for 10 min, the spores were stimulated with 0.001 mM GR24 at the fifth minute, and the data were recorded every 30 s [35].

### Nanoplastic distribution, hyphal morphology, and secondary spore number

Hyphal samples from control and NP-treated (20 μg ml^−1^, pH 5.5) cultures were used to assess NP distribution, morphology, and secondary spore number. To detect fluorescent NPs, hyphae were washed in phosphate buffer (0.1 M, pH 7.2), mounted in PBS on glass slides, and examined under 535/610 nm excitation using the CLSM. For ultrastructural analysis, hyphae were processed for scanning and transmission electron microscopy (SEM and TEM). Briefly, samples were fixed in aldehydes (2.5% glutaraldehyde with or without 2% paraformaldehyde), postfixed if required, dehydrated through a graded ethanol series, and critical-point-dried (for SEM) or embedded in epoxy resin and ultrathin-sectioned (for TEM). SEM samples were sputter-coated with gold prior to observation. Observations were made using a Hitachi SU8100 SEM and an FEI Tecnai G2 Spirit Bio TWIN TEM, operating at standard voltages.

A Leica DFC450 stereomicroscope was employed to examine hyphal branching and secondary spore formation at consistent magnification across five replicates per treatment. For each treatment, in 200 randomly selected fields of view, 200 segments of hyphae (each with 400 μm in length) were chosen to count the number of secondary spores and BAS. This length ensured that most hyphae within the field of view were adequately represented. Additionally, 200 branching points where thin runner hyphae emerged from thick runner hyphae were randomly selected for measurement. At these points, the total length of the thin runner hyphae was recorded. Overlap of highly branched hyphae, a common issue that can affect measurement accuracy, was addressed by excluding the length of BAS from the statistical analysis [36]. To minimize the impact of overlapping hyphae, adjustments were made to the light intensity and microscope focus, improving visualization and ensuring more reliable measurements of the thin runner hyphae. This approach is commonly used in fungal studies to enhance clarity and ensure precise data collection [37].

### Determination of hyphal chitinase and succinate dehydrogenase activity

Chitinase and succinate dehydrogenase (SDH) activities in hyphae from control and NP-treated (20 μg ml^−1^, pH 5.5) cultures were determined using commercial assay kits (BC0820 and BC0955, Solarbio Technology, China) according to the manufacturer’s protocols. Enzyme activities were calculated as defined by the manufacturer and are expressed per gram of tissue (chitinase) or per milligram of protein (SDH) [38, 39].

### Pot experiment

#### Host plant and soil preparation

The maize (*Zea mays* L.) cultivar Zhengdan958 was used in the pot trial. The maize seeds (purchased from Hefei Hefeng Seed Co. Ltd) were surface-sterilized with 10% (v/v) H_2_O_2_, followed by thorough rinsing with ultrapure water 10 times. The sterilized seeds were then placed on seedling trays for germination. The soil used was collected from Dinghu Mountain (23°9′41″N, 112°32′36″E) in Zhaoqing City, Guangdong Province. The soil was classified as reddish red soil derived from sandy shale, with the following physicochemical properties: pH 5.9 (soil: H_2_O ratio of 1:5), total N 1.4 g kg^−1^, total P 0.20 g kg^−1^, soil organic carbon (SOC) 13.7 g kg^−1^, and available P 3.8 mg kg^−1^. After collection, the soil was air-dried and sieved through a 2 mm mesh. Essential nutrients were supplemented to each kilogram of soil as follows: 200 mg N ((NH_4_)_2_SO_4_), 10 mg P (KH_2_PO_4_), 50 mg Mg (MgSO_4_·7H_2_O), 5 mg Zn (ZnSO_4_·7H_2_O), 5 mg Mn (MnSO_4_·H_2_O), and 2 mg Cu (CuSO_4_·5H_2_O). Pots were filled with 1.5 kg of the prepared soil. Each pot was thinned to one seedling after emergence. Each pot was inoculated with 600 randomly selected secondary spores reproduced in the Petri-dish culture treated with 0 or 20 μg ml^−1^ NPs. There were three replicates for each treatment. The pot experiment was conducted in a greenhouse under natural sunlight, with a temperature of 25°C and relative humidity of 70%–90%. To ensure uniformity, pots were randomly positioned and regularly rotated.

#### Plant sample preparation, determination of shoot biomass, photosynthetic rate, shoot P concentration, mycorrhizal colonization, and root activity

After the maize had grown for 40 d, the photosynthetic rate was measured in three mature leaves using an LI-6400XT portable photosynthesis system (LI-COR, USA) during mid-morning hours (9–11 a.m.). Samples were harvested on Day 56, washed with ultrapure water, and separated into shoots and roots. The shoots were weighed and dried at 105°C for 1 h, then at 65°C until constant weight was achieved. Biomass and plant P concentrations were determined according to a previously published method [40]. The root samples were chopped into 1 cm root segments, mixed evenly, and divided into two parts. One part was stored in 70% ethanol, and the colonization rate was determined according to a previously published method [41]. Root activity was determined using the TTC (2,3,5-triphenyltetrazolium chloride) reduction method with a commercial assay kit (BC5275, Solarbio, China).

#### RNA extraction of root samples and quantitative PCR (q-PCR) analysis

Gene expressions in plant roots mediating P acquisition were quantified. Subsamples of plant roots stored at −80°C were used to determine six genes mediating root P uptake (i.e. *Pht*1;1 to *Pht*1;6, detailed in Table S2). The total RNA in plant tissues was extracted using phenol-chloroform reagent (Invitrogen, USA). The extracted RNA was converted to complementary DNA (cDNA) using a Transcriptor First Strand cDNA Synthesis Kit (TaKaRa, Japan). To quantify gene expressions, qPCR was carried out with TB Green Premix Ex Taq II (TaKaRa, Japan) and a MyiQ real-time PCR detection system (ABI QuantStudioTM 6, Thermo, USA). The *Alpha tubulin4* served as a reference gene [42]. cDNA amplification was carried out using a PCR procedure with 95°C for 300 s, 40 cycles of 95°C for 10 s, 57°C for 30 s, and 72°C for extension. The relative expression of each gene was determined as previously described [43].

#### Determination of soil available P concentration, acid phosphatase activity, hyphal length density, and 16S ribosomal RNA (rRNA) gene copy numbers

After removing the topsoil (2 cm), the remaining soil was homogenized, separated into two batches, and stored at 4°C and −80°C, respectively. Available P was extracted with 0.03 M ammonium fluoride–hydrochloric acid and determined using the molybdenum blue method [44]. Soil acid phosphatase (S-ACP) activity was determined using a BC0140 kit (Solarbio Technology, Beijing, China) following the manufacturer’s instructions. Approximately 0.1 g of soil sample, passed through a 0.6 μm nylon sieve after natural air-drying, was weighed. To this, 0.05 ml of toluene was added. The mixture was shaken for 15 min. Then, the reaction reagent was added, and the mixture was incubated at 37°C for 24 h. After incubation, the corresponding reagent was added. The sample was centrifuged at 8000 rpm for 10 min at 25°C. Finally, the absorbance of the supernatant was measured at 660 nm. S-ACP activity was defined as 1 nmol of phenol released per gram of soil per day at 37°C as one enzyme activity unit [45]. Hyphal length density (HLD) was determined using the membrane filter technique and the gridline intercept method [46]. Briefly, 5 g of dry soil samples were added to 50 ml of distilled water in a pulverizer and stirred for 30 s. After filtration and centrifugation, the liquid was vacuum-filtered through a 0.45 μm filter membrane. After staining with trypan blue, 30 fields of view were observed using the gridline intercept method at ×200 magnification and then converted to HLD [46].

To assess the impact of NPs on AM fungal colonization and, subsequently, on rhizosphere microbiota, total bacterial abundance was quantified using real-time PCR targeting the 16S rRNA gene. Rhizosphere DNA was extracted from NP-treated and control samples using the FastDNA SPIN Kit for Soil (MP Biomedicals, USA). Real-time qPCR was performed on an ABI QuantStudioTM 6 system (Applied Biosystems, USA) using universal bacterial primers 519F (5′-GTGCCAGCMGCCGCGGTAA-3′)/907R (5′-CCGTCAATTCMTTTRAGTTT-3′) with SYBR Green Master Mix (TOYOBO, Japan). The thermal profile included: initial denaturation at 95°C for 5 min; 40 cycles of 95°C (15 s), 60°C (30 s), and 72°C (1 min). Melting curve analysis (65°C–95°C, 0.5°C increments) confirmed amplification specificity, with no-template controls demonstrating the absence of contamination. Gene copy numbers were normalized to soil dry weight [42].

#### Soil DNA extraction and P-related gene relative abundance analysis

We used the FastDNA Spin Kit for DNA Isolation from MP Biomedicals to extract DNA from soil samples. Approximately 0.5 g of soil was used for the extraction according to the manufacturer’s instructions. Concentrations of the extracted DNA were determined using the NanoDrop 2000 (Thermo Scientific, USA), and the DNA was stored at −20°C for future use. DNA concentrations were first diluted to 20 ng μl^−1^, and quantitative microbial element cycling (QMEC) was used to quantify the relative abundance of nine genes associated with P cycling and 16S rRNA genes as previously described [47]. A high-throughput qPCR chip, SmartChip MyDesign Chip (Takara Biomedical Technologies, Clontech), and a SmartChip real-time fluorescent quantitative PCR system (WaferGen Biosystems USA) were used to determine the levels of gene expression. The program was set to denaturation at 95°C for 10 min, denaturation at 95°C for 30 s for 40 cycles, annealing at 58°C for 30 s, and extension at 72°C for 30 s. Detection and Ct values were obtained for each sample using Canco software. When the Ct value was 0 or >31, the gene was considered undetected; the mean value of the gene detected in all three technical replicates was calculated as the Ct value of the gene in the corresponding sample. The relative value (R) for each gene (including the 16S rRNA gene) was calculated from the Ct value using the formula: R = 10^((31 − Ct)/3.333). The relative abundance of a target gene was then determined by normalizing its relative value to that of the 16S rRNA gene: Relative Abundance = R_target/R_16S. [47].

### Statistical analysis

One-way analysis of variance (ANOVA) followed by Tukey’s honestly significant difference (HSD) test (*P* < .05) was used to compare means across treatments. Other indicators were analyzed using *t-*tests. The relative abundance of P-cycling-related microbial functional genes was log10-transformed prior to analysis. Figures were generated using Origin 2022. All data are expressed as mean ± standard deviation (SD).

## Results and discussion

### Nanoplastics impeded spore germination and reproduction via surface attachment

We asked whether NPs could be adsorbed onto the spore surface during the initial precolonization phase, and we clearly observed such adsorption (Fig. 1a and b). Also, the adsorption was highly dependent on NP concentration and pH in the culture medium, in that higher levels of NP concentration and pH led to greater adsorption (Fig. 1b and Fig. S3). NP internalization into spores was also observed, although the number of plastic particles was limited (Fig. S4). The surface of AM fungal spores in our experiment was irregular and rough, with undulations and protrusions (Fig. S5). These topographical variations create micro-spaces that entrap NPs.

For spore germination, the NP treatment (20 μg ml^−1^) resulted in a general reduction in germination rates compared with the control, and the reduction was more pronounced (−48.5%) on the early stage (i.e. on the fourth day) (Fig. 1d). This initial suppression suggests that NPs primarily interfere with the early stages of spore activation, possibly by disrupting the perception of external signals or the initial physiological processes required for germination [48]. Indeed, we show that NPs on spore surfaces interfered with the spore perception of GR24 (a synthetic and model SL used to trigger AM fungal spore germination), in that GR24 addition to the NP-treated spores failed to trigger the Ca^2+^ spiking intensities comparable to the nontreated ones (Fig. 1e). AM fungal spore germination requires signal molecules from the plant side (i.e. the root-secreted SLs), which are perceived by the spore to trigger intracellular Ca^2+^ spiking followed by hyphal emergence [49]. Our results of reduced Ca^2+^ spiking (Fig. 1e) suggest the NPs could physically block receptor sites or alter membrane properties, thereby disrupting signal transduction. This is consistent with the findings that nanoparticles can interact with cell membrane receptors (e.g. epidermal growth factor receptor and integrins), thereby interfering with their subsequent signal transduction [50].

For the size of secondary spores, the NP treatment (20 μg ml^−1^) significantly reduced their diameter from 88.5 to 77.6 μm (Fig. 1c) and decreased the proportion of large spores (>70 μm) from 72.7% to 54.7% (Fig. S6). The plausible reasons for the size reduction include the following: (i) because NPs can reduce hyphal activities [23], the P transfer to host plants can be hindered so that the rewarding pathway (carbon transfer from roots to AM fungi) is restricted (known as a fair, two-way transfer of resources [51]), leading to compromised AM fungal reproduction (smaller secondary spores). (ii) The surface-attached NPs blocked the hydration of spores, resulting in smaller spores. Similar hydration suppression has been observed in the spores of a fern (*C. pteridoides*) treated with NPs, which also led to a reduction in spore size [22]. The size of AM fungal spores indicates their ability to germinate and search for new hosts, because larger spores contain more energy (lipid and carbohydrate storage) [52], allowing them to persist longer and support more hyphal elongation during host search [53, 54]. Additionally, larger spores possess thicker cell walls, which provide better resistance against both abiotic and biotic stressors [55].

### Nanoplastics inhibited hyphal expansion via interrupting organelle integrity

**Figure 2:**
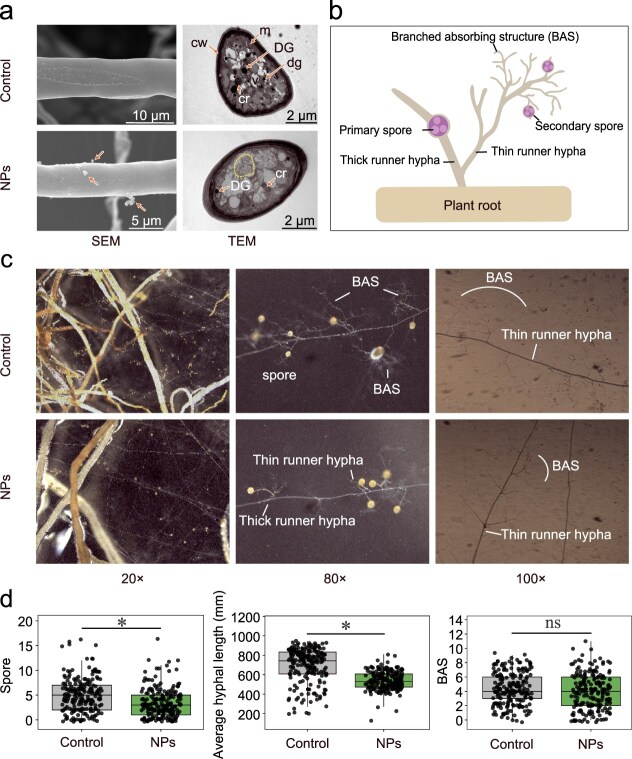
Morphological responses of extraradical hyphae to NPs. (a) SEM (scanning electron microscopy) and TEM (transmission electron microscopy) images of extraradical hyphae exposed to 0 (control) or 20 μg ml^−1^ NPs. (b) Schematic diagram of AM fungal hyphal structures, including branched absorbing structures (BAS), primary/secondary spores, and thick/thin runner hyphae. (c) Stereomicroscopy images of hyphal networks, showing spores, BAS, and runner hyphae under control and NP-treated conditions. (d) Quantitative analysis of hyphal length, spore counts, and BAS numbers in the control and NP-treated groups. Data were obtained from five culture plates per treatment (*n* = 5), with a total of 200 microscopic fields of view at the same magnification examined across replicates. In the boxplots, the horizontal line marks the median, and the box edges show the 25th and 75th percentiles. Asterisks indicate significant differences between treatments (*t-*test, *P* < .05).

Following spore germination, NPs continued to impair the establishment of the symbiosis during hyphal growth and expansion during the colonization phase. We suspected that NPs should attach to the hyphal surfaces of AM fungi, similar to spores. Indeed, SEM images show that NPs agglomerated on hyphal surfaces, forming clusters resembling grape bunches (Fig. 2a, arrows). In contrast, hyphae without NPs exhibited smooth surfaces (Fig. 2a, control).

Within hyphae, TEM images further show that hyphal vesicle and cytoplasmic boundaries began to blur under NP stress (20 μg ml^−1^). Severe plasmolysis was observed (Fig. 2a, yellow dashed circle). The number of opaque granules (og), osmophilic globules (OGs), and mitochondria (m) was significantly reduced, accompanied by numerous cavities within these organelles and shrinkage of the cell membrane (Fig. 2a). In comparison, organelle integrity was maintained in hyphae without NPs, in that numerous OGs were enclosed by a tight-fitting membrane and OGs were inside vacuoles. Also, plenty of mitochondria in the cytoplasm were clearly observed (m in Fig. 2a). Loss of organelle integrity could probably be caused by reactive oxygen species (ROS) generated under NP stress, driving organelles’ dissolution and internal structural degradation [23]. Other studies also showed that silver nanoparticles could penetrate cell membranes, induce ROS production, and disrupt fungal cellular structures [12]. Although the physicochemical properties of NPs differ from those of metal nanoparticles [56], our results revealed that NPs have a similar disruptive effect on the hyphal cell structure.

For the hyphal structure, NPs reduced the average length of thin runner hyphae (Fig. 2b), although the number of BASs per unit length remained unchanged (Fig. 2c and d). The reduction in thin runner hyphal length suggests a potential decrease in nutrient acquisition capacity [57], because they are responsible for nutrient uptake and secondary spore production [58, 59]. Indeed, we observed a reduction in the production of secondary spores following exposure to NPs (Fig. 2d). The reduced length of the thin runner hyphae could be due to the compromised mitochondria (Fig. 2a), which are responsible for energy production for hyphal growth [60]. Also, the reduction of OGs was an essential factor hindering hyphal development and reproduction, as OGs store building materials for synthesizing chitin, which is the building block of hyphal and spore cell walls [61]. Furthermore, fewer OGs in the hyphae under NP stress (Fig. 2a) can reduce the P and Ca transfer from the AM fungus to the plant, as the OGs store high concentrations of P and Ca to be transported to the plant [62].

**Figure 3:**
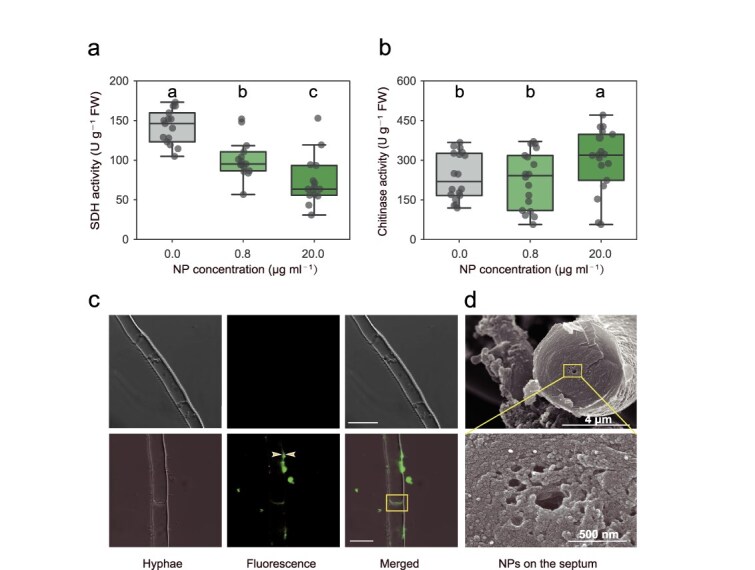
Physiological and structural responses of AM fungi to NPs. (a, b) Succinate dehydrogenase (SDH) and chitinase activities in extraradical hyphae exposed to 0 (control), 0.8, and 20 μg ml^−1^ NPs, *n* = 3 culture plates, with 15 hyphal samples measured per treatment. In the boxplots, the horizontal line marks the median, and the box edges show the 25th and 75th percentiles. Lowercase letters indicate significant differences among different concentrations (ANOVA, Tukey’s HSD test, *P* < .05). (c) Confocal microscopy (CLSM, scale bar = 10 μm) reveals NP internalization patterns, with arrows highlighting dual-layer fluorescence demonstrating NP distribution on both inner and outer hyphal walls, and boxes indicating NP enrichment at the forming septum, and (d) SEM images of NPs deposited on the surface of the septum of a hypha.

Because SDH can indicate mitochondrial activity, we also measure its activity. We show that 0.8 and 20 μg ml^−1^ of NPs reduced SDH activity by 28.6% and 48.2%, respectively, compared to the control (Fig. 3a). These significant declines align with the reduced number of mitochondria (Fig. 2a) and further confirm that NPs damaged hyphal structure (Fig. 2d). Also, 20 μg ml^−1^ of NPs led to an increase in chitinase activity (Fig. 3b), which breaks down chitin-rich cell walls [39]. It clearly suggests NPs promoted the hyphal aging, leading to a higher level of cell wall degradation. Such a degradation could be caused by the NP entry, transport, and accumulation at the hyphal septum, accelerating hyphal aging and inducing premature septum formation (Fig. 3c and d). Consequently, these septa act as barriers that restrict further NP migration in the hyphae. On the other hand, AM fungi may adapt to or recover from NP stress over multiple growing seasons. The adaptation and recovery are possibly due to (i) NP aggregate formation [23] and microbe-facilitated NP degradation/surface modification, e.g. biofilm [5], reducing NP toxicity, (ii) upregulation of tolerance-related gene expression [23], and/or (iii) hyphal capture of NPs [23] and quick renewal of hyphae [63].

### Spores reproduced under nanoplastics have less symbiotic capability in soil

**Figure 4:**
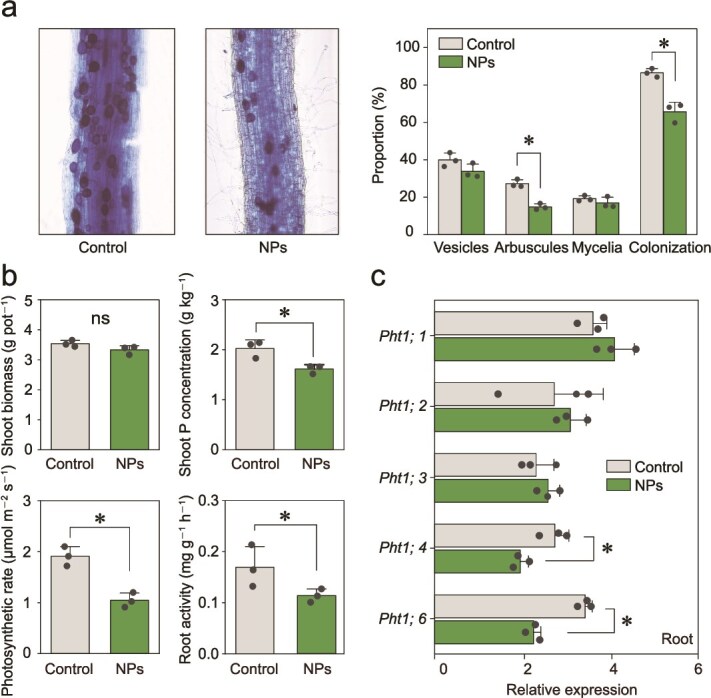
Effects of NP-treated AM fungi on plant colonization, growth performance, and phosphorus acquisition. (a) The root segments showing AM fungal colonization structures (hyphae, vesicles, and arbuscules) and colonization rates. Control, inoculated with normal spores; NPs, inoculated with NPs-treated spores. (b) Plant physiological parameters include shoot biomass, shoot phosphorus concentration, photosynthetic rate, and root activity. Asterisks indicate significant differences between treatments (*t*-test, *P* < .05). (c) Relative expression levels of *Pht* family phosphate transporter genes (*Pht*1;1 to *Pht*1;6) in maize roots. Control and NP treatments were compared using a *t-*test with a significance level of *P* < .05. All data are presented as means ± SD (*n* = 3).

Because we found that NPs reduced spore germination and the size of secondary spores, compared to the treatment without NPs (Figs 1e and 2d), we further asked whether the colonization ability of these smaller secondary spores would be affected when grown in soil. Our results show that the smaller spores exhibited significantly lower colonization rates (−24.1%) and fewer arbuscule structures (−45.5%) in maize roots, compared with the spores of normal sizes, although there was no difference for the rates of vesicles and hyphae (Fig. 4a). Arbuscule structures within root cells are the most critical fungal structures, mediating resource exchange between the plants and AM fungus [64]. The reduced colonization ability could be attributed to two reasons. First, the weakened perception of symbiotic signals (Fig. 1e) likely delayed or diminished spore germination, crippling the initial launch of the symbiotic program. Second, the reduction in spore size (Fig. 1c) implies diminished energy reserves, directly limiting the hyphal growth potential and the spore’s ability to forage for a host root, thereby reducing its chances of successful colonization. Similarly, a previous study found that the larger the AM fungal spores, the higher the germination rates [65].

Even when the smaller spores overcame the NP barriers and successfully colonized the roots, their subsequent benefits in supporting plant P acquisition were severely compromised, compared with the normal spores. Specifically, during the post-colonization phase, the plants inoculated with smaller spores had lower (−20.3%) shoot P concentrations than those inoculated with normal spores. Such P reduction likely contributed to the 45.0% decline in photosynthesis (via impaired ATP synthesis) and 33.7% decrease in root activity (Fig. 4b). The compromised P acquisition was related to the 45.5% reduction in arbuscule formation (Fig. 4a) because arbuscules are the primary interface transporting P from AM fungi to plants [66]. Furthermore, P transporter genes in maize (Zm*PHT*1;4 and Zm*PHT*1;6) were also significantly downregulated under NPs stress (Fig. 4c). Zm*PHT*1;4 mediates direct root P uptake, and Zm*PHT*1;6 participates in the mycorrhizal P uptake pathway [67]. The downregulation of Zm*PHT*1;4 and Zm*PHT*1;6 further exacerbated P deficiency, demonstrating a direct correlation between reduced arbuscule formation and decreased P uptake.

**Figure 5:**
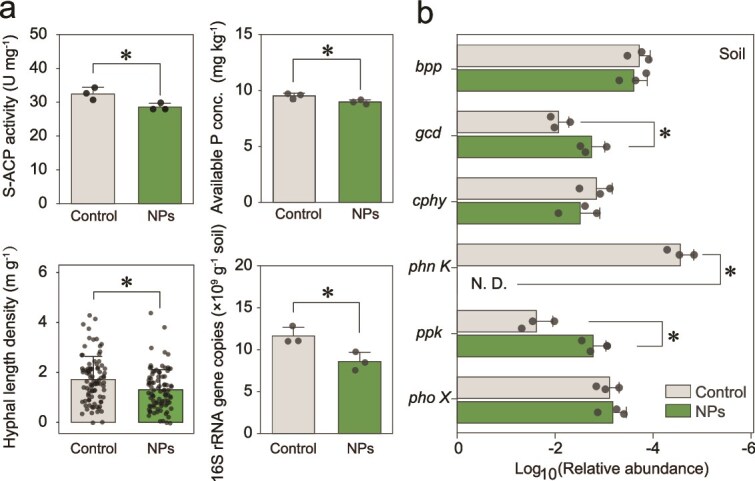
Effects of NP-treated AM fungi on soil phosphorus mobilization and functional gene expression. (a) Parameters associated with AM fungal phosphorus mobilization include acid phosphatase activity (S-ACP), soil available P concentration, hyphal length density, and total bacterial abundance (16S rRNA gene copies). (b) Relative abundance (log_10_ transformed) of key functional genes involved in P cycling. Control, inoculated with normal spores; NPs, inoculated with NPs-treated spores. Asterisks indicate significant differences (*P* < .05) between the two treatments (*t-*test).

Despite significant reductions in P uptake and photosynthesis, there were no shoot-biomass differences when inoculated with secondary spores reproduced under NP stress (Fig. 4b). As a proportion of shoot biomass, photosynthate is transported from shoots to roots, supporting AM fungi and the associated bacteria [68–70]. However, in the present study, the plant may maintain photosynthate in the shoots for its own growth rather than allocate to the AM fungus because the belowground partnership was inefficient, including decreases in AM fungal colonization rate (Fig. 4a), root activities (Fig. 4b), HLD, soil enzyme activities, and bacterial abundance (Fig. 5a).

In soil, the observed decline in P mobilization was further corroborated by the decrease of soil available P (−5.7%), significant downregulation of key P-cycling genes (*ppk*, *phnK*, *gcd*), reduction of bacterial abundance (−26.2% of 16S rRNA gene copies), and decrease in acid phosphatase activity (−11.9%) (Fig. 5b). These results indicate that the reduced carbon allocation to AM fungi not only limited hyphal exploration but also suppressed the broader microbial P-mining activity in the rhizosphere.

Regarding soil bacteria, the significant decrease in HLD (Fig. 5a) could lead to reduced production of hyphal exudates, which are carbon sources attracting PSB in the hyphosphere [71]. PSB is essentially needed for AM fungal P uptake because AM fungi have genetic constraints to mineralize organic P [20, 72]. The secondary spores (reproduced under NP stress) had reduced ability in producing hyphae, as evidenced by the declined HLD (Fig. 5a). The declined HLD resulted in reduced hyphal surface area, amount of exudates as carbon sources, and travel distance for bacteria; NPs could also physically obstruct the hyphal surface, limiting the availability of both hyphal exudates and attachment sites for bacteria. Consequently, PSB colonization and activity decreased, as shown by the reduction in 16S rRNA gene copies and acid phosphatase activity (Fig. 5a). This reflects that NPs interfered with the carbon-based communication among the plant, AM fungus, and bacteria. The ecosystem implications of this disruption are multifaceted. Reduced P uptake efficiency may compromise plant performance, particularly in P-limited soils where the mycorrhizal pathway dominates P acquisition [73]. Additionally, the loss of hyphal biomass may have downstream effects on soil carbon sequestration, because AM fungal networks are critical for soil aggregation and organic matter stabilization [74]. These findings underscore the complex interplay between NPs, AM fungi, and soil microbes, highlighting their critical roles in soil P cycling and plant nutrition.

## Conclusion

In summary, this study provides a comprehensive mechanistic understanding of how NPs interrupt the critical symbiotic partnership between plants and AM fungi across the entire colonization process. We demonstrate that NPs impair spore germination and symbiotic signal perception during the precolonization phase, damage hyphal organelles and accelerate senescence during the colonization phase, and ultimately reduce hyphal length density and P mineralization capacity during the postcolonization phase. These cascading effects lead to significantly compromised plant P uptake and overall performance. Our findings reveal that the ecotoxicity of NPs extends beyond individual organisms to disrupt essential plant-fungal interactions, thereby posing a significant threat to terrestrial ecosystem functioning and agricultural sustainability. To extrapolate our findings into natural environments, we need to consider other factors that possibly affect NP behavior, including soil buffering (chemical and physical), accumulation of exudates, continuous plastic degradation, microbial community dynamics, and their combined and long-term effects.

## Supplementary Material

SI_NPS_AMF_2026_4_17_clean_wrag101(1)

## Data Availability

The data of this work are available in the Supplementary Information and deposited at https://doi.org/10.6084/m9.figshare.31341787.
