# Supplementary material for: Nanoplastics interfere with plant–mycorrhizal communication and limit plant growth
Source: ISME J. 2026 Apr 23;20(1):wrag101. doi: 10.1093/ismejo/wrag101 (PMC13271390; doi:10.1093/ismejo/wrag101)
Supplement: SI_NPS_AMF_2026_4_17_clean_wrag101(1) [file si_nps_amf_2026_4_17_clean_wrag101(1).docx]

**Supplementary Information**

Nanoplastics interfere with plant-mycorrhizal communication and limit plant growth

Han Hao Li^1, 2^‡, Xun Wen Chen^1, 2^‡, Ming Ge Xing^1, 2^, Yong Xi Zhao^1, 2^, Miao Miao Zhang^1, 2^, Quan Ying Cai^1, 2^, and Hui Li^1, 2*^

^1^ Department of Ecology, College of Life Science and Technology, Jinan University, Guangzhou 510632, Guangdong Province, China

^2^ MOE Key Laboratory of Tumor Molecular Biology, College of Life Science and Technology, Jinan University, Guangzhou 510632, Guangdong Province, China

‡ These authors contributed equally to this work.

^*^ Corresponding author: Hui Li (e-mail: tlihui@jnu.edu.cn)

Address: Department of Ecology, College of Life Science and Technology

The 2^nd^ Science and Engineering Building, Main Campus

Jinan University, Guangzhou, China

This file includes:

Extended Methods

Supplementary Figures S1–S6

Supplementary Tables S1 and S2

# Supplementary Methods

## Root organ culture establishment

In detail, each Petri dish was evenly divided into a root compartment (RC) and a hyphal compartment (HC), separated using a thin plastic barrier (90 × 15 mm) (Fig. 1a). Twenty mL of modified MSR medium (Table S1) was placed in the RC. Then, approximately 5 cm of hairy roots together with disinfected AM fungal spores were placed on the MSR medium. The hairy roots are carrot (*Daucus carota* L.) roots transformed with T-DNA from a tumor-inducing plasmid, while the AM fungal species is *Rhizophagus irregularis* (DAOM197198, Biosystematics Research Center, Ottawa, Canada). The AM fungal spores were disinfected using Tween 80 and chloramine T solution, rinsed with 1% (w/v) streptomycin sulfate and 0.5% (w/v) gentamicin sulfate solution before inoculating into the RC.

After placing roots and fungal spores in the RC, each Petri dish was sealed with Parafilm and cultured in an incubator at 27℃ in the dark. The growth status of the carrot roots was regularly checked, and roots crossing the plastic barrier were trimmed to ensure none were present in the HC, while AM fungal hyphae were allowed to grow into the HC. After 60 d, AM fungal hyphae could be observed crossing the plastic barrier and entering the HC. The Petri dishes with well-grown AM fungal hyphae were collected for subsequent use.

## Characteristics of nanoplastics

The NPs used were positively charged amine-functionalized polystyrene (PS-NH_2_) because positively charged NPs exhibited more pronounced toxicity toward AM fungi [1]. To visually track their deposition, the NPs were fluorescence-labeled using the non-toxic fluorescein isothiocyanate, which can be visualized under excitation wavelengths of 535/610 nm (Huge Biotechnology Co., Ltd., Shanghai, China). Such fluorescence-labeled PS-NH_2_ particles were commonly used as the model plastic particles representing positively charged NPs [1, 2]. The morphology of NPs was characterized using transmission electron microscopy (FEI-Tecnai G2 Spirit TWIN, Thermo) and scanning electron microscopy (SEM, SU8100, Hitachi). The size and Zeta potential (ζ) of NPs were determined with dynamic light scattering (DLS) using a Zetasizer Nano ZS90 instrument (Malvern Instruments Ltd). Before the experiment started, all NPs were transferred to a dialysis bag (1 kDa) containing deionized water for 7 d to remove any impurities, with the deionized water being replaced every 12 h. Characterization of the NPs (PS-NH_2_) is shown in Fig. S1.

## Fluorescent dye leakage and quenching experiments

Since the fluorescent dye may leach and/or quench, we conducted dye-leakage experiments for 7 d and quenching experiments for 28 d (covering the experimental periods) to ensure that leakage and quenching would not significantly affect NP visualization. The fluorescence-labeled NPs mixed with liquid MSR culture medium were filtered through the 3 kDa molar mass cut-off filter units each day to obtain filtrate, but exclude NPs. The process was repeated on each sample for 7 d [3]. On day 7, the filtrates were observed using a confocal laser scanning microscope (CLSM) (LSM 880 with AiryScan, Carl Zeiss), and no fluorescent signal was recorded at wavelengths of 535/610 nm, indicating that dye leakage was negligible during the period.

For the quenching test, NPs were placed in Petri dishes containing liquid MSR medium, wrapped with aluminum foil, and incubated in darkness at 27°C (the same conditions for AM fungal cultivation below). The fluorescence intensity of the NPs was continuously measured every 3 d to monitor for quenching. In detail, on days 0, 3, 6, 9, 12, 15, 18, 21, 24, and 28, the medium containing NPs was ultrasonicated, and 0.2 mL was collected. The samples were then observed at 470 nm using a CLSM with an identical detector gain. The average fluorescence intensity was obtained using Zen 3.0 software (blue edition; Carl Zeiss). We found that the fluorescence intensities remained stable during 28 days (Fig. S2), suggesting quenching was limited.

The above two results suggest that either fluorescence leakage or quenching was negligible, thereby validating the fidelity of the NP-tracking methodology.

# **Supplementary Figures**


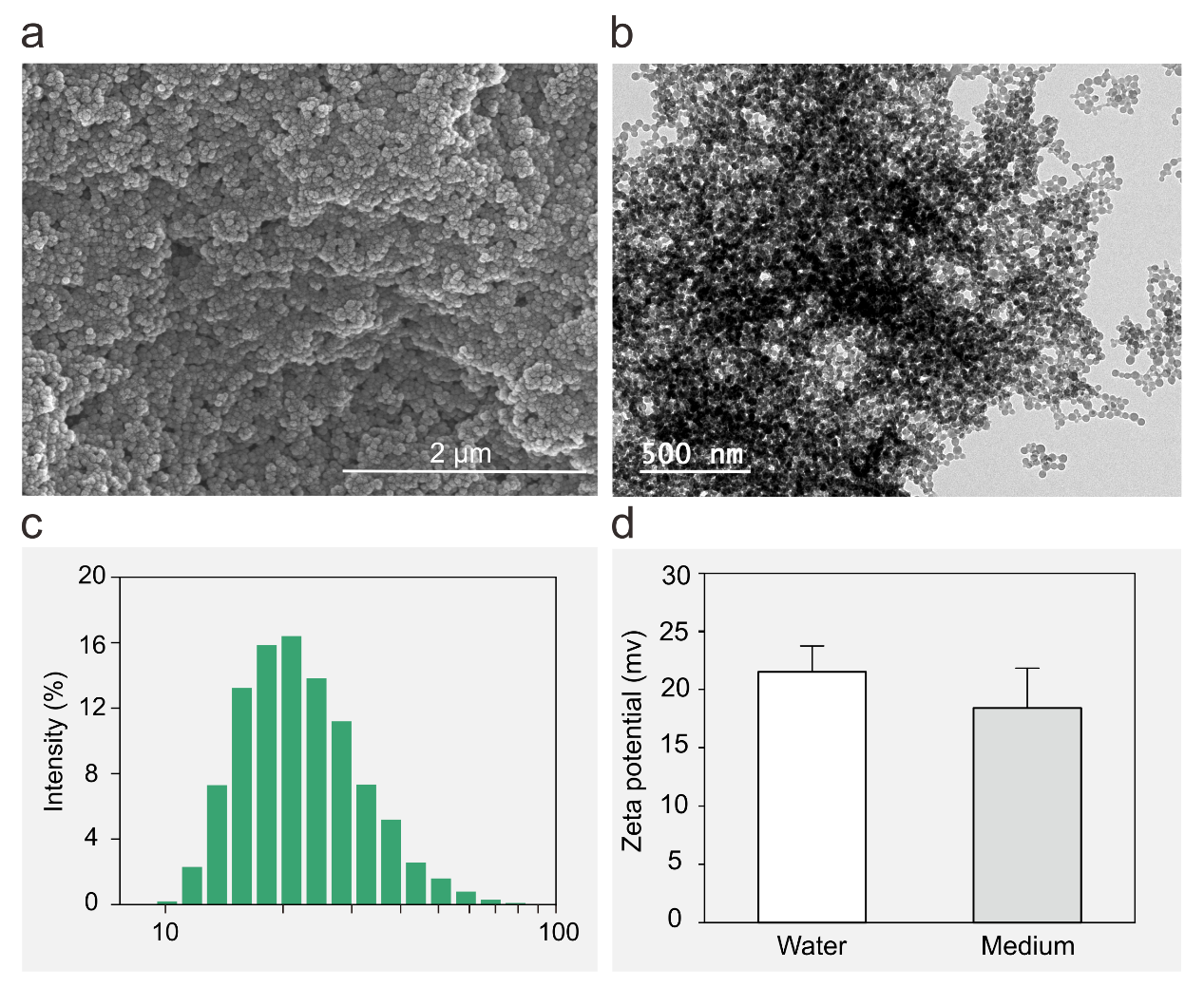


### Fig. S1. Characteristics of the NPs used in the present study.

(a) SEM image and (b) TEM image of NPs; (c) Size distribution of NPs as assessed by dynamic light scattering (DLS) in deionized water; (d) Zeta potentials of NPs in sterile deionized water (pH = 7.3) and MSR medium (pH = 5.5). Data are mean ± S.D. (n = 3).


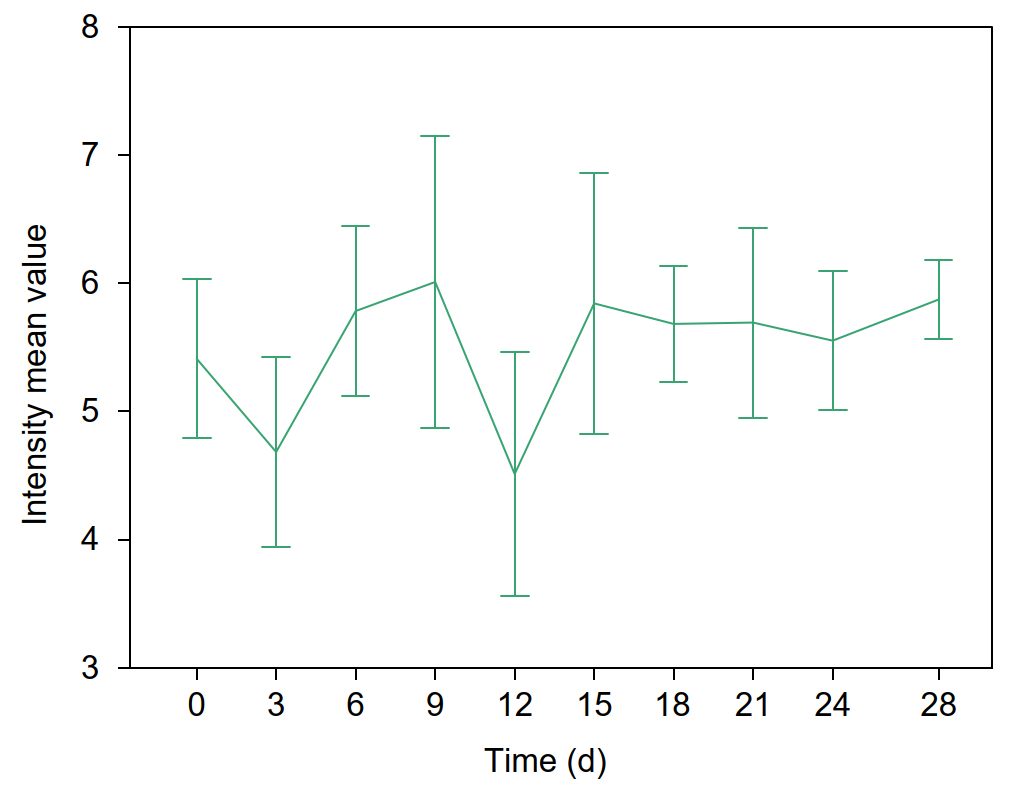


### Fig. S2. Average fluorescence intensity changes of NPs over 28 d.

Measurements were conducted every three days. The average intensity was measured using CLSM with respective wavelengths. Data are mean ± S.D. (n = 3).


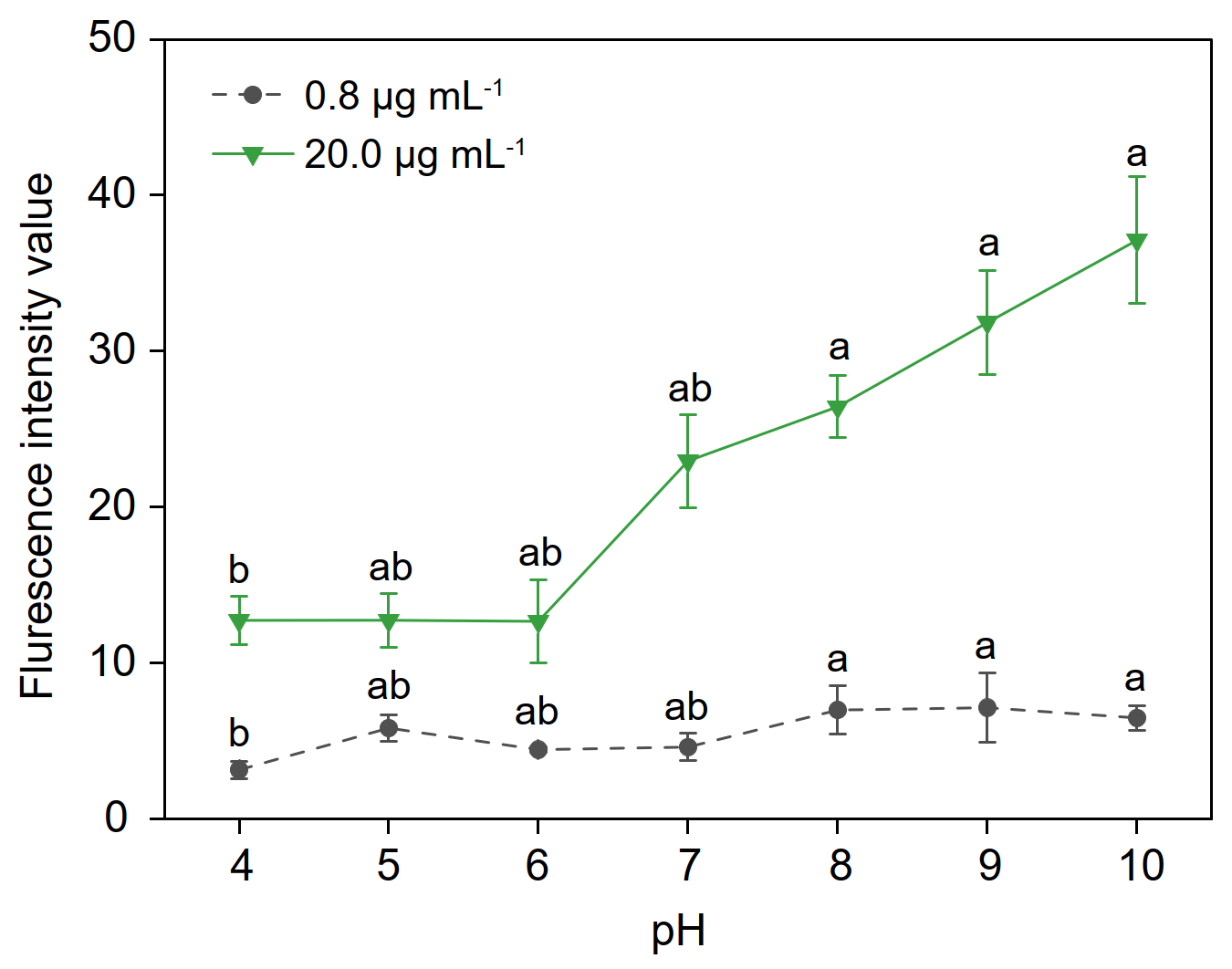


### Fig. S3. Fluorescence intensity of NPs adsorbed on spores.

Different lowercase letters on error bars indicate significant differences at the significance level of 0.05 (ANOVA followed by Tukey's HSD test). Data are mean ± S.D. (n = 3).


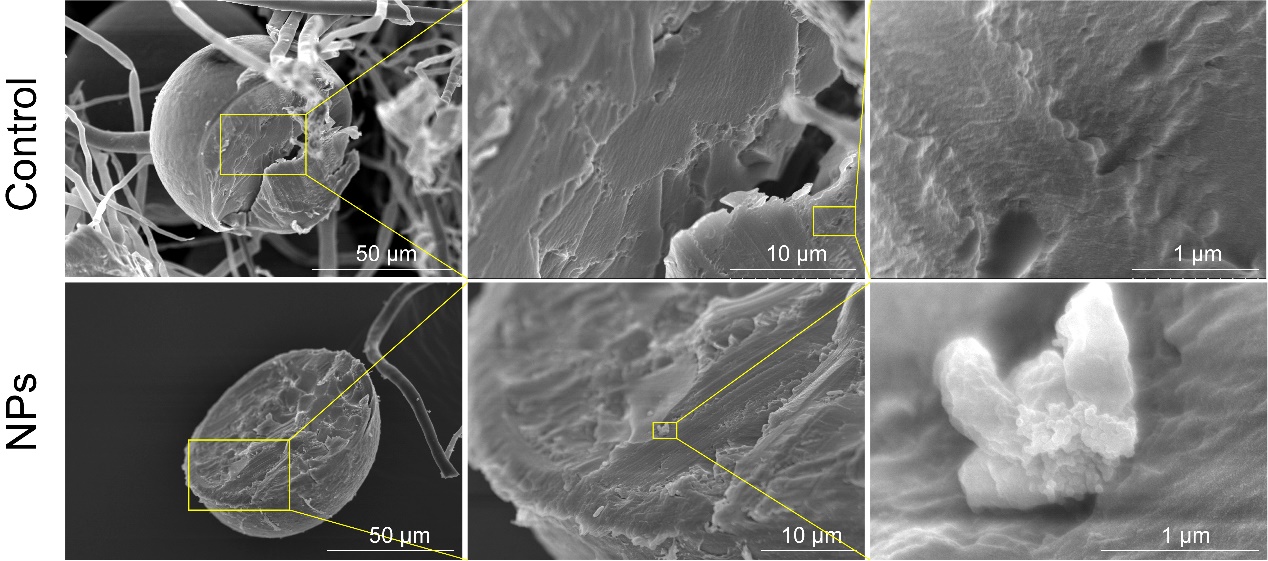


### Fig. S4. Scanning electron microscopic images of NPs inside the spores.

The images were taken after the treatment with 20 μg ml^－1^ NPs at pH = 5.5.


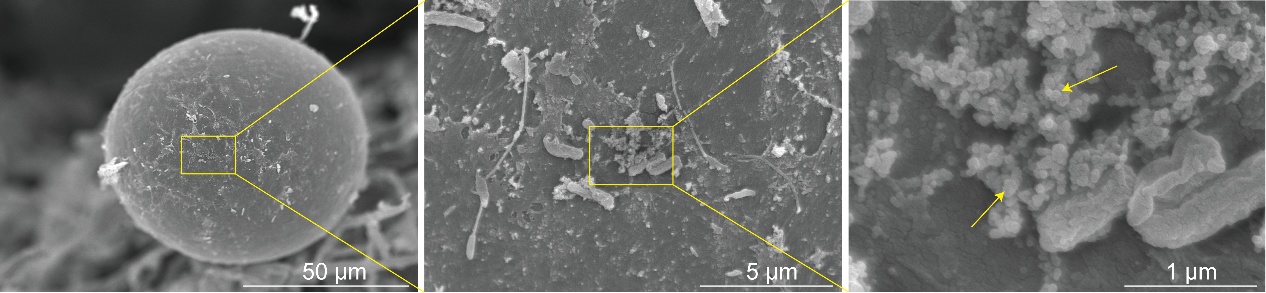


### Fig. S5. SEM images of NPs (yellow arrow) adsorbed on the surface of a spore.


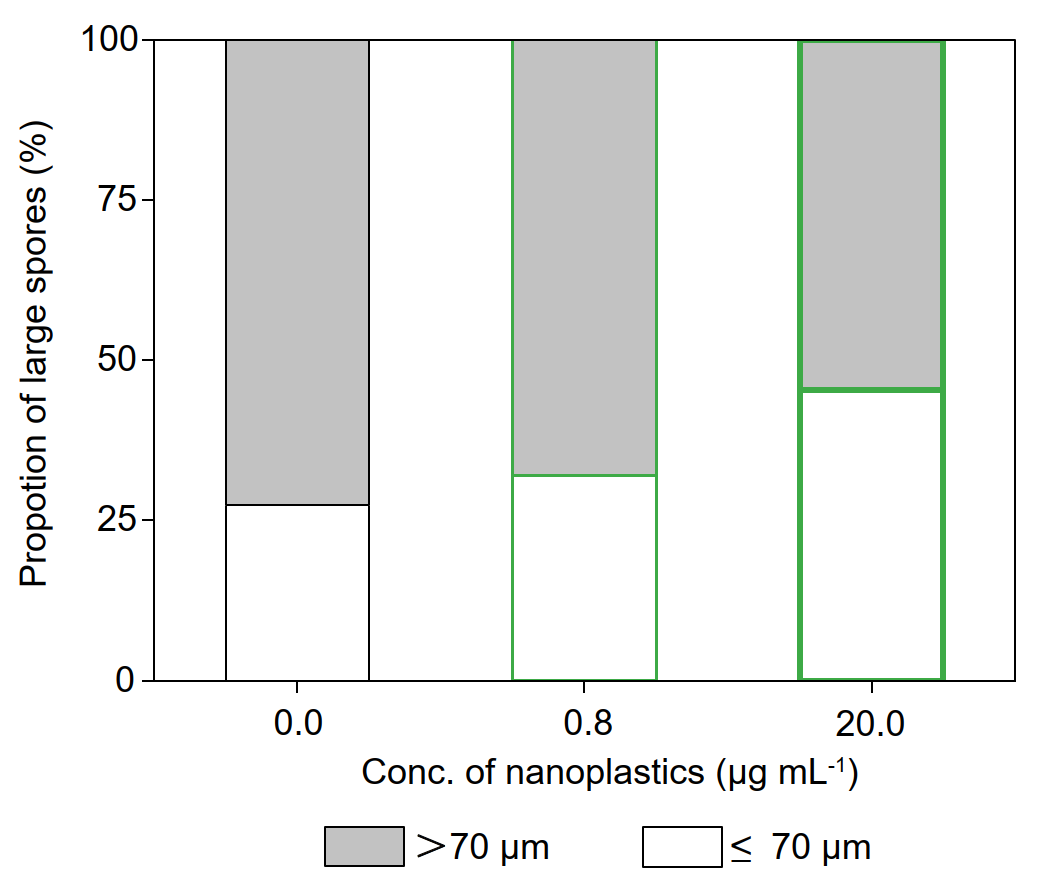


### Fig. S6. Proportion of secondary spores of different sizes.

The secondary spores were reproduced in growth medium without or with NPs.

# **Supplementary Tables**

### Table S1. MSR culture medium used in the root organ experiment.

| Chemical |  | Final concentration (mg L^−1^) | |  | |
| --- | --- | --- | --- | --- | --- |
| MgSO_4_·7H_2_O |  | 731 |  | |  |
| KNO_3_ |  | 80 |  | |  |
| KCl |  | 65 |  | |  |
| KH_2_PO_4_ |  | 4.8 |  | |  |
| Ca(NO_3_)_2_·4H_2_O |  | 288 |  | |  |
| NaFeEDTA |  | 8 |  | |  |
| Na_2_MoO_4_·2H_2_O |  | 0.0024 |  | |  |
| KI |  | 0.75 |  | |  |
| MnCl₂·4H_2_O |  | 6 |  | |  |
| ZnSO_4_·7H_2_O |  | 2.65 |  | |  |
| H₃BO₃ |  | 1.5 |  | |  |
| CuSO_4_·5H_2_O |  | 0.13 |  | |  |
| 4-Hydroxy-D-phenylglycine |  | 3 |  | |  |
| Inositol |  | 50 |  | |  |
| Vitamin B1 |  | 20 |  | |  |
| Vitamin B6 |  | 20 |  | |  |
| Vitamin B3 |  | 50 |  | |  |

### Table S2. Genes and primers derived from RT-qPCR analysis.

| Target gene |  | Primer sequence |
| --- | --- | --- |
| *ZmPhtl; l* | forward | 5' GACCCAGATGGTGTAGAATCGAACAT 3' |
|  | reverse | 5' TCACTTACTTTCCCGCCTATAACACACA 3' |
| *ZmPhtl;2* | forward | 5' GTCTGGTGAGGCTGAAGACTCAGAGG 3' |
|  | reverse | 5' ACATGATAGCCCACCATGTGCAGTGC 3' |
| *ZmPhtl;3* | forward | 5' TGTTTCCGTTCTGTCTGGTGCTTGTG 3' |
|  | reverse | 5' TCCCGACGGTGACCTCCGATTATTTA 3' |
| *ZmPhtl;4* | forward | 5' GAGACCCAGATGGTGTAGAGAATCG 3' |
|  | reverse | 5' CATCAAAACACAGCCAGGGTTGACT 3' |
| *ZmPhtl;6* | forward | 5' CGGACGTGAGCAAGGATGACAA 3' |
|  | reverse | 5' GGATTCCACACCCCCTGTGTAGT 3' |
| *Alpha tubulin4* | forward | 5' GCTATCCTGTGATCTGCCCTGA 3' |
|  | reverse | 5' CGCCAAACTTAATAACCCAGTA 3' |

# References

1. Li HH et al. Arbuscular Mycorrhizal Fungus Alleviates Charged Nanoplastic Stress in Host Plants via Enhanced Defense-Related Gene Expressions and Hyphal Capture. *Environ Sci Technol* 2024;**58**:6258–6273. https://doi.org/10.1021/acs.est.3c07850

2. Liu M-Y et al. Nitrogen Forms Regulate the Response of Microcystis aeruginosa to Nanoplastics at Environmentally Relevant Nitrogen Concentrations. *ACS Nano* 2024;**18**:11828–11836. https://doi.org/10.1021/acsnano.4c00739

3. Xiao F et al. Do polystyrene nanoplastics have similar effects on duckweed (*Lemna minor* L.) at environmentally relevant and observed-effect concentrations? *Environ Sci Technol* 2022;**56**:4071–4079. https://doi.org/10.1021/acs.est.1c06595
